# Understanding proteome quantification in an interactive learning module on Google Cloud Platform

**DOI:** 10.1093/bib/bbae235

**Published:** 2024-07-23

**Authors:** Kyle A O’Connell, Benjamin Kopchick, Thad Carlson, David Belardo, Stephanie D Byrum

**Affiliations:** Center for Information Technology, National Institutes of Health, 9000 Rockville Pike, Bethesda MD, 20892, United States; Health Data and AI, Deloitte Consulting LLP, 1919 N Lynn St, Arlington VA, 22209, United States; Center for Information Technology, National Institutes of Health, 9000 Rockville Pike, Bethesda MD, 20892, United States; Health Data and AI, Deloitte Consulting LLP, 1919 N Lynn St, Arlington VA, 22209, United States; Center for Information Technology, National Institutes of Health, 9000 Rockville Pike, Bethesda MD, 20892, United States; Health Data and AI, Deloitte Consulting LLP, 1919 N Lynn St, Arlington VA, 22209, United States; Google Cloud, 1900 Reston Metro Plz, Suite 1400, Reston VA, 20190, United States; Department of Biochemistry and Molecular Biology, University of Arkansas for Medical Sciences, 4301 W. Markham St., Little Rock, AR, 72205, United States; Arkansas Children's Research Institute, 1 Children's Way, Little Rock, AR, 72202, United States; Department of Biomedical Informatics, University of Arkansas for Medical Sciences, 4301 W. Markham St, Little Rock, AR, 72205, United States

**Keywords:** proteomics, Google Cloud Platform, mass spectrometry

## Abstract

This manuscript describes the development of a resource module that is part of a learning platform named ‘NIGMS Sandbox for Cloud-based Learning’ https://github.com/NIGMS/NIGMS-Sandbox. The overall genesis of the Sandbox is described in the editorial NIGMS Sandbox at the beginning of this Supplement. This module delivers learning materials on protein quantification in an interactive format that uses appropriate cloud resources for data access and analyses. Quantitative proteomics is a rapidly growing discipline due to the cutting-edge technologies of high resolution mass spectrometry. There are many data types to consider for proteome quantification including data dependent acquisition, data independent acquisition, multiplexing with Tandem Mass Tag reporter ions, spectral counts, and more. As part of the NIH NIGMS Sandbox effort, we developed a learning module to introduce students to mass spectrometry terminology, normalization methods, statistical designs, and basics of R programming. By utilizing the Google Cloud environment, the learning module is easily accessible without the need for complex installation procedures. The proteome quantification module demonstrates the analysis using a provided TMT10plex data set using MS3 reporter ion intensity quantitative values in a Jupyter notebook with an R kernel. The learning module begins with the raw intensities, performs normalization, and differential abundance analysis using limma models, and is designed for researchers with a basic understanding of mass spectrometry and R programming language. Learners walk away with a better understanding of how to navigate Google Cloud Platform for proteomic research, and with the basics of mass spectrometry data analysis at the command line.

This manuscript describes the development of a resource module that is part of a learning platform named ``NIGMS Sandbox for Cloud-based Learning'' https://github.com/NIGMS/NIGMS-Sandbox. The overall genesis of the Sandbox is described in the editorial NIGMS Sandbox [1] at the beginning of this Supplement. This module delivers learning materials on the analysis of bulk and single-cell ATAC-seq data in an interactive format that uses appropriate cloud resources for data access and analyses.

## INTRODUCTION

Proteins are large and complex molecules that play a critical role in biological systems. Proteins do most of the work in cells and are required for structure, function, and regulation of the body’s tissues and organs. High resolution mass spectrometry is the technology used to analyze these complex molecules. However, mass spectrometers do not sequence amino acids, rather they measure mass-to-charge ratios with a limited detection range. In bottom-up proteomics, proteins are digested into small peptides in order to allow the instruments to detect the peptides and then the mass spectra are matched against a database of theoretically digested proteins to identify the peptide sequence and ultimately the protein. The amount of ions for a given peptide detected is translated into the peptide abundance and it is the sum of abundance values for peptides belonging to the same protein that is then used for proteome quantification.

**Figure 1:**
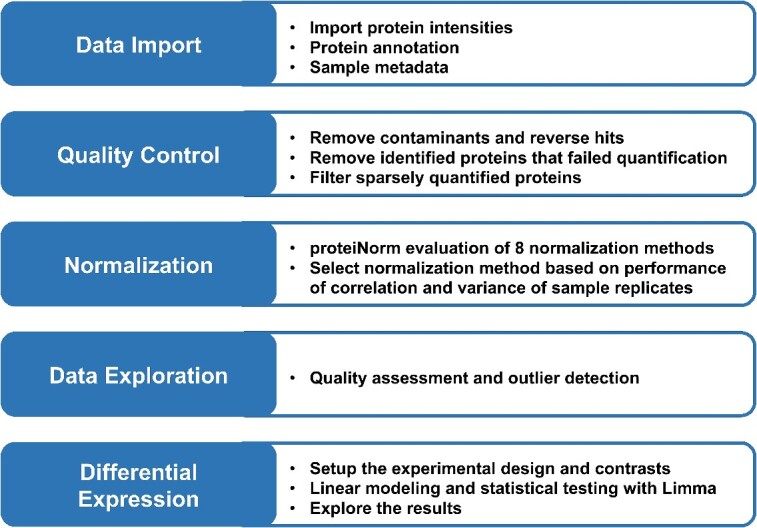
Proteome quantification workflow. A matrix of protein intensities, protein annotation, and sample metadata is imported, the data is checked for quality, data is normalized, and differential analysis is performed.

Data is acquired on a mass spectrometer using either data dependent acquisition (DDA) or data independent acquisition (DIA). In DDA the top abundant peptides injected into the mass spectrometer are analyzed whereas for DIA methods, all peptides within a mass window are detected. DDA experiments can utilize multiplexed samples using Tandem Mass Tags (TMT) reporter ions, which provide highly quantitative values for sample comparisons. TMT methods are limited in the number of samples allowed in an experiment due to batch effects between two multiplexed experiments. For DIA, the samples are not multiplexed but rather the MS2 spectra are multiplexed to provide higher coverage of peptide detection. Depending on the mass spectrometry workflow used to generate the data, the quantitative values will change.

Data preprocessing of mass spectrometry data is crucial to performing differential abundance analysis. The learning module we developed will discuss key terms of proteomics, how to interpret MS data quality, and perform differential analysis. The module built using Jupyter notebooks is designed for users getting started with R programming. The cell blocks containing R code for the various plots are meant to be modified by the user in order to understand how the visualizations are generated in base R. The code for the statistical analysis follows the statistical design models from the *Limma* R package [2]. The complete functionality is included in an R package called *proteoDA*, which can be downloaded from https://github.com/ByrumLab/proteoDA [3]. Learners will gain the following skills upon completion of the module:

1. Basic understanding of mass spectrometry.
2. Proteomics normalization.
3. Statistical designs for differential abundance analysis.
4. Basic R programming.
5. Data Visualization.

**Table 1:** Sample metadata used for proteome quantification. The data includes three cell lines MCF10A (normal), MDA-MB-231 (triple negative breast cancer), and HCC1937 (triple negative breast cancer with BRCA1 mutant) with either hydroxyurea treatment (TR) or untreated (UT) samples. The samples were prepared in two multiplexed TMT10plex batches.

ID	Sample Name	TMT batch	Group
1	MCF10A_UT1	1	MCF10A_UT
2	MCF10A_UT2	1	MCF10A_UT
3	MCF10A_UT3	1	MCF10A_UT
4	MDAMB231_UT1	1	MDAMB231_UT
5	MDAMB231_UT2	1	MDAMB231_UT
6	MDAMB231_UT3	1	MDAMB231_UT
7	HCC1937_UT1	1	HCC1937_UT
8	HCC1937_UT2	1	HCC1937_UT
9	HCC1937_UT3	1	HCC1937_UT
10	MCF10A_TR1	2	MCF10A_TR
11	MCF10A_TR2	2	MCF10A_TR
12	MCF10A_TR3	2	MCF10A_TR
13	MDAMB231_TR1	2	MDAMB231_TR
14	MDAMB231_TR2	2	MDAMB231_TR
15	MDAMB231_TR3	2	MDAMB231_TR
16	HCC1937_TR1	2	HCC1937_TR
17	HCC1937_TR2	2	HCC1937_TR
18	HCC1937_TR3	2	HCC1937_TR

## MATERIALS AND METHODS

### Setting up the environment

To start with this tutorial, users need to set up their Google Cloud Platform environment by launching a Jupyter notebook instance within Vertex AI. All instructions for setting up the compute environment are available within the NIH Cloud Lab GitHub repository (https://github.com/STRIDES/NIHCloudLabGCP). Participants in the NIGMS Sandbox program are granted access to cloud resources through the NIH Cloud Lab Program. Once inside Google Cloud Platform, users will navigate to the VertexAI platform and spin up a VertexAI notebook instance with an R kernel. We recommend users to choose an n1-standard-4 machine type (4 vCPUs and  16 GB RAM).

Users clone all module data and Jupyter notebooks from the GitHub repository (https://github.com/NIGMS/Proteome-Quantification). Once the environment has been set up and the repository cloned, users will open the .ipynb file and confirm the R kernel is selected. The cells in the notebook will provide proteomic term definitions, R code to install packages and perform functions, visualizations with explanations for each step in proteome quantification, and R code to perform differential analysis.

### Proteomics dataset

The data associated with this Proteome Quantification learning module is available from ProteomeXchange PXD025238. The mass spectrometry data was generated from triple negative breast cancer cell lines MDA-MB-231 (BRCA1 wild-type), HCC1937 (BRCA1 mutant), and non-tumorigenic epithelial breast MCF10A cells digested with trypsin, labeled using tandem mass tags (TMT) 10-plex isobaric labeling reagent (Thermo), and analyzed by Orbitrap Eclipse Tribrid mass spectrometer (Thermo) using DDA multi-notch MS3 parameters [4]. Proteins were identified and reporter ions quantified by searching the UniprotKB *Homo sapiens* database using MaxQuant with MS3 reporter ion intensities.

**Figure 2:**
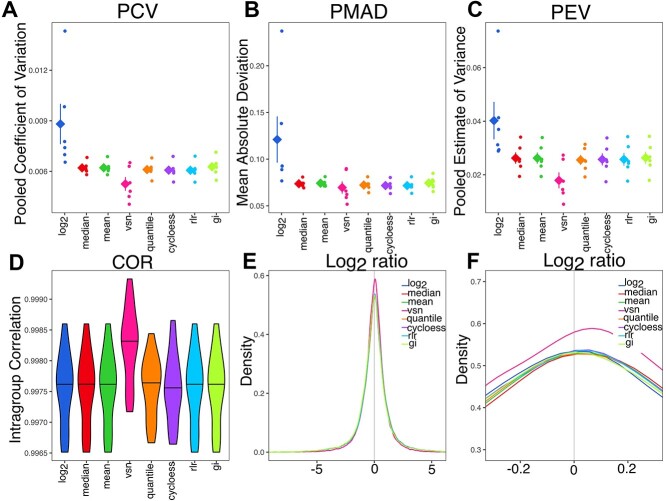
Evaluation of normalization methods. *ProteiNorm* evaluates eight normalization methods including log2 transformation (unnormalized), median, mean, variance stabilizing normalization (VSN), quantile, cyclic loess, robust linear regression (rlr), and global intensity (gi). The sample group variance is evaluated by A) pooled coefficient of variation, B) mean absolute deviation, and C) pooled estimate of variance. D) The correlation plots include multiple dots per group for all the pairwise intragroup correlations. E) The log2 ratio evaluates the normalization method skewing of the fold change values when comparing sample group conditions. F) is a zoomed in view of E.

## RESULTS

The Proteome Quantification cloud-based training module provides a Jupyter Notebook with an R kernel where the user will acquire a basic understanding of mass spectrometry data types, preprocessing considerations, normalization, differential analysis, and the R programming language. The overall workflow includes (i) data import, which includes the sample metadata, protein intensities, protein annotation such as UniprotID, Gene name, protein description, (ii) quality control, which filters contaminants and sparsely quantified proteins, (iii) Normalization is evaluated and performed to remove technical bias, (iv) data exploration to assess quality and sample outliers, and (v) differential expression analysis using *limma* models (Fig. 1).

The example data provided with the notebook includes quantitative values for three cell lines including MCF10A (normal epithelial), MDA-MB-231 (triple negative breast cancer), and HCC1937 (triple negative breast cancer with BRCA1 mutation). Cells were treated with hydroxyurea to induce a DNA damage response and investigate the BRCA1 pathway. Triplicate samples of each cell line with and without treatment were multiplexed into two TMT10plex batches (Table 1). The resulting TMT batch effect is shown with the principle component analysis provided in the tutorial.

**Figure 3:**
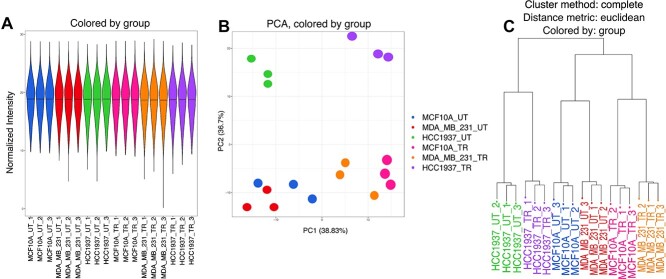
Quality control evaluation of normalized data. The normalized data is evaluated for outlier samples and missing values as visualized by A) violin, B) principle component analysis (PCA), and C) clustered dendrograms. The sample groups have similar mean normalized intensities and are separated by principle component 1 and 2 in the PCA plot and show separation in the clustered dendrogram.

**Figure 4:**
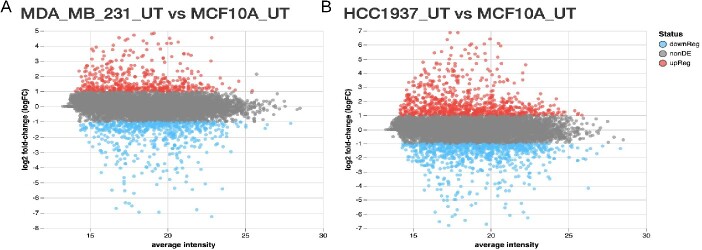
Mean-difference plot from differential analysis. The differential analysis for A) MDA-MB-231 or B) HCC1937 triple negative breast cancer compared to MCF10A epithelial cell line without hydroxyurea treatment results are shown where the x-axis is the log2 average intensity of proteins, the y-axis is the log2 fold change, and the color represents an adjusted *P*-value <.05 (red is up-regulated and blue is down-regulated).

After the data is imported, the quality is assessed and normalized using *proteiNorm*, which evaluates eight normalization methods including log2 transformation, median, mean, variance stabilizing normalization (VSN), quantile, cyclic loess (Ritchie et al), robust linear regression (rlr), and global intensity (gi) (Fig. 2A-F) [5]. The performance of each method is evaluated for intragroup variance and correlation by the following metrics: pooled coefficient of variation, mean absolute deviation, pooled estimate of variance, correlation, and log2 ratio. The normalization method with the lowest variance and the highest correlation within the group replicates should be selected for analysis. The log2 ratio plot should be centered on zero and not introduce bias toward up- or down-regulation of proteins. In the example data set, the normalization method with the lowest variance across sample group replicates and the highest replicate correlation is VSN normalization. Therefore, the data for differential analysis is normalized using VSN. Both the MS3 reporter ion intensities before and after normalization is provided in the data example file with the learning module.

Once the normalization method has been selected, the data is normalized and ready for analysis using linear models but first the data is checked for sample outliers and the amount of missing values. The normalized data is displayed using a violin plot to check the overall distribution of proteins in each sample, a principle component analysis (PCA) for detection of outlier samples, and a clustered dendrogram of the sample similarity (Fig. 3A-C). The first two principle components (PC) from PCA are shown in Fig. 3B and indicate 38.83% and 36.7% variability explained in PC1 and PC2, respectively. The clustered dendrogram was generated using the complete method with the euclidean distance metric (Fig. 3C). The Jupyter notebook describes the types of linear models available from the R *limma* package. The example utilizes the ‘no intercept’ model in the example so that the sample group comparisons can be specified as needed for the differential analysis [6]. The statistical results are visualized using a mean-difference plot highlighting the log2 average normalized intensities on the x-axis, the log2 fold change on the y-axis, and the color represents the adjusted *P*-value <..05 (Fig. 4). The title of the mean difference (MD) plots indicate the order the contrasts were run in the *limma* model such that the proteins with a fold change >2 will be highlighted in red (positive values) and correspond to the first group listed in the title. For example, ‘MDA_MB_231 versus MCF10A’ indicates positive fold changes are up-regulated in the MDA_MB_231 group compared to the MCF10A sample group. The log2 average intensity value allows users to quickly identify proteins that are lower abundant and possibly at the limit of detection by the mass spectrometry. These lower abundant proteins will sometimes have more missing values across samples and/or more variability in their intensity values.

## DISCUSSION

Proteomics is a challenging field for introductory users because it requires knowledge of mass spectrometers and how the data is acquired so that the results can be accurately interpreted. Mass spectrometry technology is advancing at a rapid pace by increasing the amount of data that is acquired and our ability to identify lower abundant proteins including post-translational modifications on those proteins. The computational power necessary to analyze more and more complex protein data will only grow in scale over time. The computational resources available via cloud computing (in this case Google Cloud) allow for scalability when it comes to analyzing these complex protein mixtures.

The workflow presented in the Proteome Quantification training module is meant to serve as an introduction to Cloud-based computing, R programming language, key terms in mass spectrometry and data acquisition, challenges in data preprocessing, and differential abundance analysis. Together with the other resources of the NIGMS Sandbox program, our module will contribute to training the next generation of biomedical researchers.

**Key Points**

- Proteome Quantification training module is an open-source cloud based workflow that introduces terms and concepts for analyzing high resolution mass spectrometry data sets.
- This module can be used to analyze protein intensity data by evaluating the data quality, performing normalization, and performing differential abundance analysis.
- The Jupyter notebook is designed is designed to be interactive and the code modified in order to understand the basics of R programming.

## Data Availability

The source code and data are publicly available through the NIH Cloud lab at https://github.com/NIGMS/Proteome-Quantification.

## Notes

**Kyle O’Connell** is a bioinformatician with Deloitte Consulting LLP. His work focuses on developing and optimizing cloud-based Omics pipelines and on genomic workflow development in Nextflow, Snakemake and WDL. His research focuses on the use of AI/ML for species delimitation.

**Ben Kopchick** is a data scientist at Deloitte Consulting LLP. His work focuses on cloud-based pipelines for medical image analysis, machine learning, and privacy-enhancing technologies.

**Thad Carlson** is a cloud engineer with Deloitte Consulting LLP that specializes in building bioinformatics oriented cloud infrastructure for the research community.

**David Belardo** is a customer engineer with Google Public Sector, helping Healthcare and Life Sciences customers adopt Cloud technologies to advance research.

**Stephanie Byrum** is an Associate Professor at the University of Arkansas for Medical Sciences. She is also the Director of the UAMS Bioinformatics core, the Director for the IDeA National Resource for Quantitative Proteomics Bioinformatics service line, and the Director of the Center for Translational Pediatric Research Proteogenomics core. Her research interests include proteogenomics and multi-omics data integration methodologies. She specializes in advanced proteomics data analysis utilizing high resolution mass spectrometry.
